# The role of the gut microbiome and its metabolites in cerebrovascular diseases

**DOI:** 10.3389/fmicb.2023.1097148

**Published:** 2023-04-14

**Authors:** Hongyu Xu, Ziyue Xu, Shengrong Long, Zhengwei Li, Jiazhi Jiang, Qiangqiang Zhou, Xiaopeng Huang, Xiaohui Wu, Wei Wei, Xiang Li

**Affiliations:** ^1^Department of Neurosurgery, Zhongnan Hospital, Wuhan University, Wuhan, Hubei, China; ^2^Brain Research Center, Zhongnan Hospital, Wuhan University, Wuhan, Hubei, China

**Keywords:** gut microbiota, gut microbial metabolites, intestinal dysbacteriosis, cerebrovascular diseases, microbiota-gut-brain axis

## Abstract

The gut microbiome is critically involved in maintaining normal physiological function in the host. Recent studies have revealed that alterations in the gut microbiome contribute to the development and progression of cerebrovascular disease *via* the microbiota-gut-brain axis (MGBA). As a broad communication network in the human body, MGBA has been demonstrated to have significant interactions with various factors, such as brain structure and function, nervous system diseases, etc. It is also believed that the species and composition of gut microbiota and its metabolites are intrinsically linked to vascular inflammation and immune responses. In fact, in fecal microbiota transplantation (FMT) research, specific gut microbiota and downstream-related metabolites have been proven to not only participate in various physiological processes of human body, but also affect the occurrence and development of cerebrovascular diseases directly or indirectly through systemic inflammatory immune response. Due to the high mortality and disability rate of cerebrovascular diseases, new treatments to improve intestinal dysbacteriosis have gradually attracted widespread attention to better ameliorate the poor prognosis of cerebrovascular diseases in a non-invasive way. This review summarizes the latest advances in the gut microbiome and cerebrovascular disease research and reveals the profound impact of gut microbiota dysbiosis and its metabolites on cerebrovascular diseases. At the same time, we elucidated molecular mechanisms whereby gut microbial metabolites regulate the expression of specific interleukins in inflammatory immune responses. Moreover, we further discuss the feasibility of novel therapeutic strategies targeting the gut microbiota to improve the outcome of patients with cerebrovascular diseases. Finally, we provide new insights for standardized diagnosis and treatment of cerebrovascular diseases.

## 1. Introduction

Cerebrovascular diseases refer to conditions that cause brain tissue damage due to intracranial blood circulation disorders caused by various reasons (Thomas, 1996). The predominant clinical manifestations are transient ischemic attack (TIA), stroke, cerebral arteritis, and cognitive impairment (Mehanna and Jankovic, 2013; Dichgans and Leys, 2017). Stroke is the most common clinical manifestation of cerebrovascular diseases. In particular, ischemic stroke resulting from cerebrovascular diseases is the most prevalent cause. According to the current statistics, stroke caused by cerebrovascular diseases has become the second leading cause of death in industrialized countries and the most common reason for permanent acquired disability (O'Donnell et al., 2016). Therefore, increasing studies have concentrated on risk factors for cerebrovascular diseases (Boehme et al., 2017; Cipolla et al., 2018; Claeys et al., 2020). Early intervention in the associated risk factors can reduce the incidence of cerebrovascular disease. At present, hypertension, diabetes, smoking and gender have been identified as the main risk factors for cerebrovascular diseases. (Muhammad et al., 2021; Tsai et al., 2021). Simultaneously, with the application of multi-omics approaches (McCombie et al., 2019; Chen et al., 2021), numerous studies, notably the human microbiome project (HMP) and metagenomics of the human intestinal tract (MetaHIT) have emerged and provided a comprehensive reference for the composition of the human gut microbiota (Peterson et al., 2009; Arumugam et al., 2011). Since then, research has uncovered the function of microbiomes in varieties of diseases, mainly including cancer immunotherapy (Li et al., 2019), systemic inflammatory diseases (Clemente et al., 2018), and cardiovascular system diseases (Jie et al., 2017). Recent studies have revealed that the gut microbiota has evolved into an inseparable and symbiotic relationship with the host during the evolutionary process (Zou et al., 2022).

The composition of the human gut microbiome is dynamically balanced, and it also plays essentials roles in the human body: the circulating metabolism of various nutrients, the formation of the intestinal immune protection system, the promotion of the development of the nervous system (Yadav et al., 2018; Adak and Khan, 2019; Schoeler and Caesar, 2019). Once intestinal dysbacteriosis is under certain circumstances, it is a severe blow to the homeostasis of the gut microbiota and the health of the body. GBA refers to the two-way communication exchange network between the brain and gut microbiome, composed of the brain, intestines, and gut microbiota (Cryan et al., 2019). Recent studies have shown that ecological imbalances of the gut microbiota can disrupt the integrity of the intestinal barrier, allowing pathogens and toxic metabolites to invade the systemic circulation, resulting in the dysregulation of GBA. The ensuing immune system dysregulation and neuroinflammation can induce neurotoxic misfolded proteins to accumulate around neurons, eventually triggering neuronal death. At the same time, central nervous system involvement can aggravate intestinal dysbacteriosis through defective autophagy-mediated, thus forming a vicious circle mediated by defective autophagy and immune system disorders (Chidambaram et al., 2022). Many studies have indicated that intestinal dysbacteriosis has become an extremely significant risk factor for the onset and development of cerebrovascular diseases (Benakis et al., 2020a; Zhu S. et al., 2020).

The mammalian gut microbiota includes bacteria, viruses, fungi, yeasts, and bacteriophages (Rutsch et al., 2020), in which bacteria are the main components of the gut microbiome. Current research divides the gut microbiota into four main categories: Bacteroidota, Actinomycetes, Pseudomonadota, and Bacillota (Wolter et al., 2021). Communication between the gut microbiome and the brain has recently received widespread attention. The concept of GBA also has emerged (Pellegrini et al., 2020). Interactions between the brain, intestines, and gut microbiota regulate the physiological processes of the human body. It has been confirmed that nervous system diseases from early brain development to old age are closely related to GBA (Socała et al., 2021). Multiple anatomical structures, systems, and metabolic pathways are involved in establishing a bidirectional connection between the gut microbiota and the brain, such as neuroendocrine (*via* the HPA axis), neuroimmune system, and the sympathetic and parasympathetic arms of the autonomic nervous system including the enteric vagus nerve system and the immune system (Carabotti et al., 2015; Rao and Gershon, 2016), proposed the concept of GBA, which demonstrates bidirectional communication and mutual influence between the gut and brain through the gut microbiome in immune (Li et al., 2019), endocrine (Régnier et al., 2021) and neuromodulation (Quigley, 2017). With the further deepening of the study of gut microbiota, intestinal dysbacteriosis and gut microbiota metabolites are not merely risk factors. They also strongly correlate with the prognosis and treatment of cerebrovascular diseases (Osadchiy et al., 2019; Sorboni et al., 2022). This review discusses the research progress of several most common cerebrovascular disorders. The gut microbiota introduces the close relationship between cerebrovascular diseases and the gut microbiota and its metabolites. In addition, we look forward to the possible research directions in the future and provide new ideas for further research on the diagnosis and treatment of cerebrovascular diseases.

## 2. Gut microbiota and metabolites

### 2.1. Gut microbiota

The gut microbiome comprises more than 1,500 species distributed in more than 50 phyla (Gomaa, 2020). Bacteroides and firmicutes, followed by Proteus, Fusobacterium, Ciliate, Actinomycetes, and Verrucous bacteria, have been reported to be the most dominant species of the gut microbiome, accounting for 90% of the total human microbiome (Passos and Moraes-Filho, 2017). Therefore, its abundance ratio is an essential indicator of the degree of intestinal dysbacteriosis (Kuziel and Rakoff-Nahoum, 2022). The role of the gut microbiota in the human body goes far beyond its function of promoting the digestion and absorption of food. Current research has confirmed that the gut microbiota can participate in various life activities, such as behavioral cognition (Mohajeri et al., 2018), endocrine regulation (Farzi et al., 2018), and immune response (Sadler et al., 2020). The gut microbiota produces biologically active metabolites that affect many aspects of host life activity and are widely considered the largest endocrine organ in the human body (Witkowski et al., 2020). Several factors can alter the composition and function of the gut microbiome, including host genetics, diet, age (Odamaki et al., 2016), birth pattern (Nagpal et al., 2017), and antibiotics (Hasan and Yang, 2019). Among these numerous environmental factors, diet is considered the most crucial factor determining the diversity and composition of the human gut microbiota (Wu et al., 2011; David et al., 2014). Changes in the composition and function of the gut microbiota can affect intestinal permeability, digestion and metabolism, and immune responses, resulting in metabolic disorders, vascular inflammation, immune responses associated with the nervous system, and more (Al Bander et al., 2020; González Olmo et al., 2021). Therefore, current studies have shown that the gut microbiota is closely related to obesity, diabetes, hypertension, Parkinson’s disease, Alzheimer’s disease and other diseases (Durack and Lynch, 2019). Based on a series of case–control and CeVD (Cerebral small vessel disease) animal model studies, a significant correlation between cerebrovascular disease and gut microbiota has been demonstrated. It can be seen that the gut microbiota can play a role similar to that of metabolic organs, producing a series of bioactive factors through metabolic pathways that act on the host and thus affect the occurrence and development of cerebrovascular diseases (Haghikia et al., 2018).

### 2.2. Gut microbial metabolites

Gut microbial metabolites mainly come from the food the host cannot or does not have time to digest and the endogenous mucus secreted by the intestinal epithelial cells. After the action of the gut microbiota, many metabolites that are harmful or beneficial to the human body are produced, such as short-chain fatty acids (SCFA), bile acids (BA), choline metabolites, vitamins, etc. Among them, SCFA, Trimethylamine N-oxide (TMAO), lipopolysaccharide (LPS) and BA have been widely confirmed to participate in various inflammatory responses, immune responses, signaling and other processes (Martin-Gallausiaux et al., 2021; Matsushita et al., 2021), thereby affecting the occurrence and development of cerebrovascular diseases.

### 2.3. Short-chain fatty acids

SCFA is the main product of dietary fiber fermentation in the colon, and the flora that produces SCFA mainly includes anaerobes, bifidobacteria, eubacteria, streptococci, and lactobacilli (Sadler et al., 2020). Adults produce approximately 500–600 mmol of SCFA in their gut daily. Acetate, propionic acid, and butyric acid are the most abundant SCFA in the human body and the most abundant anions in the colon (Kim et al., 2022). Because acetate, butyrate and propionate in SCFA easily cross the blood–brain barrier (BBB), and SCFA has neuroactive properties and its impact on other intestinal-brain signaling pathways, including immune and endocrine systems, SCFA may be directly or indirectly involved in the occurrence and development of cerebrovascular diseases and exert its biological role (Clarke et al., 2014; Stilling et al., 2016; Wenzel et al., 2020). SCFAs possess favorable anti-inflammatory and chemopreventive properties. SCFAs are also considered as tumor inhibitors to exert anti-cancer and anti-inflammatory effects in cerebrovascular disease. Among them, the anti-cancer and anti-inflammatory effects of propionate and butyrate have been confirmed (Säemann et al., 2000; Verhaar et al., 2020). Current research confirms that SCFA is not only involved in cerebral angiogenesis but is also active in the management of complications, sequelae, and post-stroke recovery(Chen et al., 2019; Lee et al., 2020; Sadler et al., 2020; Huang Q. et al., 2022).

#### 2.3.1. Trimethylamine N-oxide

TMAO, one of the gut microbial metabolites most associated with cerebrovascular diseases, is an amine oxide produced by choline, betaine, and carnitine, which is mainly obtained through the intake of foods rich in choline, L-carnitine, and phosphatidylcholine (Ascher and Reinhardt, 2018). It is processed by the human gut microbiota, converted into trimethylamine (TMA), and then enters the liver through the portal system. It is oxidized to TMAO by Flavin Monooxygenase 3 (FMO3), and then released into the bloodstream for action. The association between TMAO levels and diseases is still controversial. TMAO has been proven to directly lead to platelet hyperreactivity and enhance thrombosis, thus increasing the risk of cardiovascular and cerebrovascular accidents (Zhu et al., 2016). The clinical research on hypertensive people in China has shown that higher TMAO level was associated with an increased risk of the first stroke. Patients in the upper tertiles had a 34% higher risk of the first stroke than those in the lowest tertiles. They also found that patients with low folate and high TMAO had the highest stroke rate (Nie et al., 2018). TMAO has been confirmed to have elevated levels in the blood of patients with atherosclerosis, hypertension, type 2 diabetes, stroke, cognitive impairment and other cardiovascular and cerebrovascular diseases (Heianza et al., 2017; Zhu Y. et al., 2020). However, a case–control study by Yin et al. (2015) has come to the opposite conclusion. They found that patients with atherosclerotic ischemic stroke and TIA episodes showed significant dysregulation in their gut microbiota and reduced levels of TMAO in their blood. There is also a lot of convincing evidence of an association between TMAO and inflammation. Chen et al. (2017) have shown that TMAO can significantly trigger oxidative stress and activate NLRP3 inflammasomes by inhibiting the SIRT3-SOD2-mitochondrial ROS signaling pathway, thereby promoting vascular inflammation leading to endothelial cell dysfunction. At the same time, it has been found that TMAO can enhance leukocyte recruitment and the expression of pro-inflammatory cytokines IL-1β, IL-18, and TNF-α, and reduce the expression of the anti-inflammatory cytokine IL-10 (Chen et al., 2017). In addition, due to individual differences in the distribution of gut microbiota, the secretion level of TMAO is also different (Kim and Jazwinski, 2018) and related to major unconscionable cerebrovascular events. Therefore, TMAO has potential research value in predicting the risk of cardiovascular and cerebrovascular diseases (Ke et al., 2018).

#### 2.3.2. Bile acids

BA is a kind of substance produced by gut microbiota mediating and regulating cholesterol metabolism, and is synthesized in the liver mainly through the action of cytochrome P450 family enzymes, such as CYP7A1, CYP27A1, CYP8B1, and CYP7B1 (Winston and Theriot, 2020). Total bile acids (TBA) in the human body can be divided into primary and secondary bile acids. Circulating BA produced in the liver and intestines can reach the brain by diffusing or crossing the BBB through BA transporters. At least 20 bile acids have been found in the brain, including conjugated and unconjugated BA (Pan et al., 2017). Therefore, the content of BA in the body is also related to the occurrence of cerebrovascular diseases. Recent studies have implicated BA in cerebrovascular disease in both positive and negative functions and are directly involved in the physiological activities and pathological processes of the brain (Weng et al., 2022). For instance, taurine deoxycholic acid (TUDCA) has been proven to be a protective BA in brain diseases with anti-apoptotic, anti-inflammatory and antioxidant characteristics (Palmela et al., 2015). In stark contrast, some BAs, such as CDCA and DCA, act as risk metabolites to alter BBB permeability by disrupting the tight junctions of rat brain microvascular endothelial cells (RBMECs; Lirong et al., 2022). Overall, BA metabolism and the BA pool are engaged in a straightforward interface between the gut microbiota and cerebrovascular disease, integral to internal environmental homeostasis.

#### 2.3.3. Lipopolysaccharide

LPS is a major component of the outer membrane of gram-negative bacteria, also known as endotoxin. The essential source of endotoxin is the death and disintegration of gut microbiota, which can form a protective barrier around bacteria to evade the action of antibiotics, acts on host cells, produces inflammatory cytokines, and causes endotoxemia and sepsis (Maldonado et al., 2016). The lipid A component of LPS is the primary pathogen-related molecular model (PAMP), which can interact with Toll-like receptor 4 (TLR4) (Ciesielska et al., 2021). When LPS is transferred from the intestinal tract to circulation, LPS forms a complex with LBP binding protein, and LBP can bind to CD14 on monocytes. This may lead to the production of pro-inflammatory cytokines, such as TNF-α, IL-1 and IL-6 (Sun et al., 2016). Recent studies have confirmed that inflammation is essential in developing cerebrovascular diseases, especially stroke. It is also related to the pathophysiological process of ischemia and the overall outcome after stroke (Anrather and Iadecola, 2016). Therefore, LPS is involved in the occurrence and development of stroke.

## 3. Regulation of gut microbial metabolites on interleukin

The gastrointestinal (GI) tract is considered the largest immunological organ in the body, having a central role in regulating immune homeostasis. The Human GI tract contains approximately 100 trillion bacteria, making it an important site of interaction between microorganisms and the host immune system (Rooks and Garrett, 2016). The host’s immune system dynamically balances anti-inflammatory and pro-inflammatory cytokines by interacting with the microbiota to regulate the action of effector cells and immune cells (Takiishi et al., 2017). Interleukin is a critical cytokine family which participates in many processes, such as the maturation, activation, proliferation, and regulation of immune cells, and also participates in many physiological and pathological reactions of the body. Some gut microbial metabolites such as SCFA, LPS, and BA have been elucidated in the related mechanism of an interleukin-mediated inflammatory immune response. Following dysregulation of gut microbial homeostasis, there is a massive release of intestinal inflammatory factors such as helper T-type (Th)1, Th17 and interleukin IL-6. The release of inflammatory factors results in altered intestinal permeability, barrier dysfunction and transit from the peripheral blood to the BBB. Ultimately, they act on the cerebrovascular system and take a pivotal role in the development, progression and prognosis of cerebrovascular disease.

### 3.1. Regulation of the SCFA on interleukin

SCFA serves as an essential fuel for intestinal epithelial cells (IEC), regulating IEC proliferation, differentiation and the function of subpopulations. For instance, SCFA exerts influence on intestinal motility by affecting the secretion of hormones from enteroendocrine cells, enhancing intestinal barrier function as well as host metabolism (Martin-Gallausiaux et al., 2021). Recent studies have partially clarified that SCFA regulates the expression of interleukin, thus affecting gut immunity and promoting the occurrence and development of diseases. Recent studies have found that SCFA induced the activation of microbial antigen-specific TH1 cells through G-protein coupled receptors 43 (GPR 43) and activates STAT3 and mTOR, thus up-regulating transcription factor B lymphocyte-induced maturation protein 1 (Blimp-1) and finally promoting the production of IL-10 (Sun et al., 2018). Yang et al. (2020) have shown that SCFA can promote the production of IL-22 by CD4T cells and innate lymphoid cells (ILCs) through G-protein receptor 41 (GPR41) and inhibiting histone deacetylase (HDAC). At the same time, they also found that butyric acid up-regulates the production of IL-22 by promoting the expression of aryl hydrocarbon receptor (AhR) and hypoxia-inducible factor 1α (HIF1α). In addition, propionate has shown that it acts directly on γδ T cells to inhibit their production of IL-17 in a histone-deacetylase-dependent manner. Moreover, the production of IL-17 by human IL-17-producing γδ T cells from patients with inflammatory bowel disease (IBD) is regulated by propionate (Dupraz et al., 2021).

### 3.2. Regulation of the LPS on interleukin

As a common endotoxin, LPS can activate monocytes, macrophages, endothelial cells, and epithelial cells through a cell signal transduction system and synthesize and release various cytokines and inflammatory mediators (Mohammad and Thiemermann, 2020). Then it causes a series of immune responses and participates in the occurrence and development of multiple diseases (Qin et al., 2007). The primary mechanism of LPS in immune response has been thoroughly studied: When LPS is released into the blood through intestinal epithelium in a pathological state, lipopolysaccharide-binding protein (LBP) can be combined with LPS and transported to the surface of myeloid cells. When LPS is released into the blood through intestinal epithelium in a pathological state, lipopolysaccharide-binding protein (LBP) can be combined with LPS and transported to the surface of myeloid cells. MCD14 on the surface of myeloid cells binds to it, forming the LPS-LBP-CD14 triple complex. Then it was transported to the protein complex of TLR4-MD2, and the triple complex combined with TLR4 with the help of MD-2 to activate TLR4. The activated TLR4 activates the intracellular signal transduction pathway through conformational changes. Intracellularly, IL-1R-related protein kinase (IRAK) aggregates into receptor complexes through MyD88 and MyD88 adaptor protein analogs, which activates IRAK phosphorylation. Afterward, IRAK dissociates from the complex and transmits the signal to TRAF6. The activated TRAF6 can signal transduction by activating nuclear factor-κ B-induced kinase (NIK) and transforming growth factor β-activated kinase 1 (TAK1), and activating the corresponding NF-κB and mitogen-activated protein kinase (MAPK). Eventually, it causes the release of IL-1, IL-6, and TNF-α and participates in inflammatory reactions (Cohen, 2002; Kumar et al., 2022).

### 3.3. Regulation of the BA on interleukin

**Figure 1:**
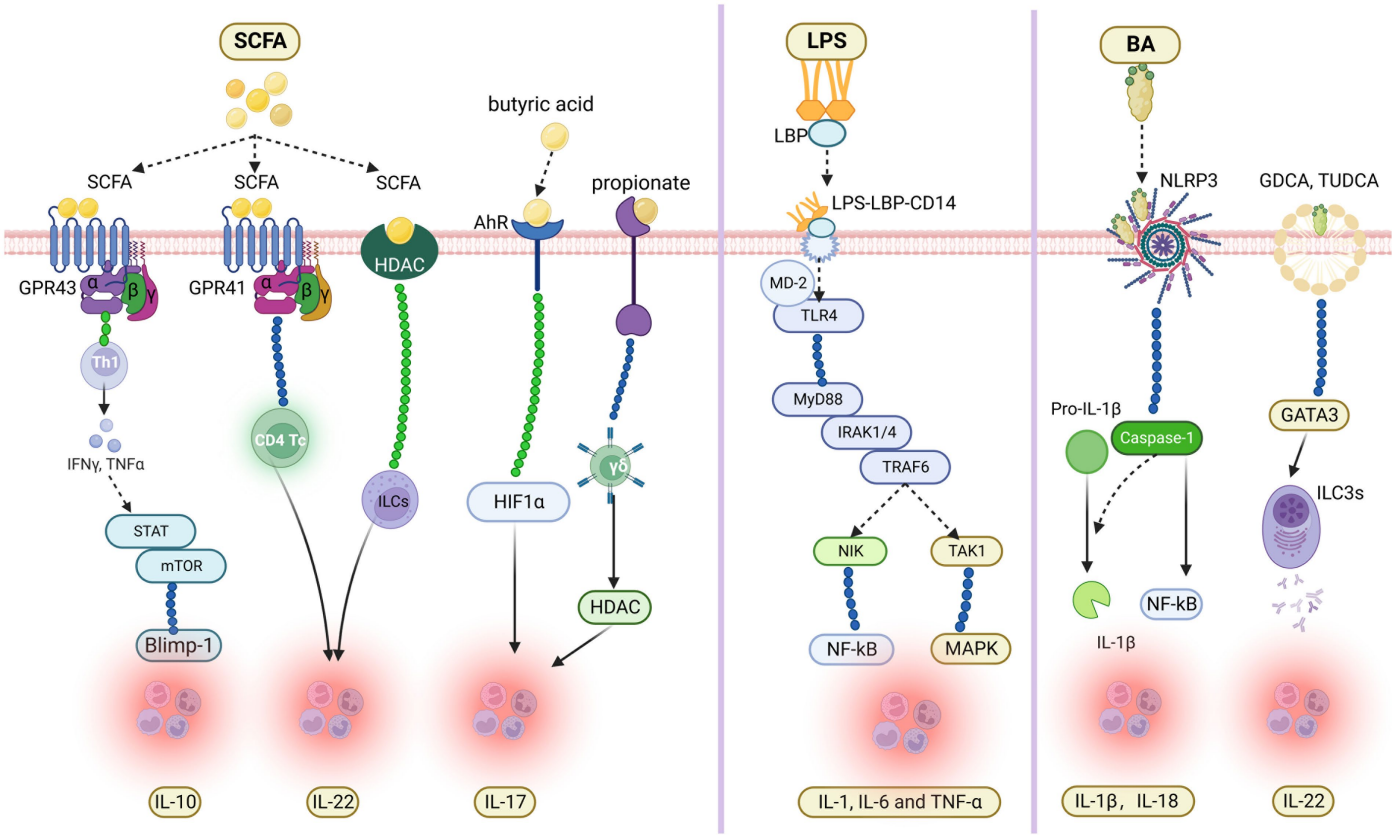
Molecular mechanism of gut microbial metabolites regulating the expression of specific interleukin in inflammatory immune response.

As a cholic acid derivative synthesized by the liver, BA is involved in many physiological and pathological processes, such as metabolism, immunity, and inflammation, playing a significant role in regulating intestinal physiological function and the disease process (Holtmann et al., 2021). Among many inflammatory cytokines, BA against NLRP3 inflammasome is not only a key mediator of host defense but also a key regulator of intestinal homeostasis (Holtmann et al., 2021). Recent studies have confirmed that BA can activate NLRP3 inflammasome to trigger the release of inflammatory factors IL-1β and IL-18 (Zhen and Zhang, 2019), promote the inflammatory process, and restore the imbalance of body homeostasis induced by PAMP (Haneklaus and O'Neill, 2015). In addition, NLRP3 inflammasome can mediate the production and release of the inflammatory factor IL-1β, and mediate cell apoptosis by triggering Caspase-1 to produce gasdermin D (Shi et al., 2017). Furthermore, the targeted preparation for NLRP3 inflammasome can effectively reduce the intestinal inflammatory response caused by BAs and is expected to reduce the occurrence of chronic autoimmune diseases such as inflammatory bowel disease. In addition, Glycodeoxycholic acid (GDCA) and TUDCA have also been proven to induce group 3 internal lymphoid cells (ILC3s) to promote the secretion of IL-22 by up-regulating GATA3 expression (Figure 1).

## 4. Gut microbiota and metabolites in cerebrovascular diseases

### 4.1. Stroke

Stroke has become a global health problem, the second leading cause of death and the third leading cause of disability (Hossmann, 2006; Kalaria, 2018). According to a systematic analysis of the worldwide burden of disease published in The Lancet, stroke became the leading cause of death from the disease in China in 2017 (Zhou et al., 2019). In humans, stroke is classified as ischemic or hemorrhagic based on the underlying neuropathology. Numerous studies have found that the gut microbiota acts on the homeostasis of the human environment through metabolic pathways and immune responses, affecting the occurrence and development of stroke (Huang and Xia, 2021; Xu et al., 2021; Peh et al., 2022).

Atherosclerotic cerebral infarction is one of the most common causes of stroke worldwide (Weinberger, 2005). Atherosclerotic cerebral infarction has been shown to be strongly correlated with TMAO. Its main mechanisms are (a) TMAO can partially increase the expression of atherosclerotic scavenger receptors CD36 and A (SRA) in macrophages, hinder cholesterol transport, and promote macrophage and foam cell formation. On this basis, mitogen-activated protein kinase (MAPK) and NF-κB signaling pathways promote endothelial inflammatory response (Seldin et al., 2016; Zhang X. et al., 2020). (b) TMAO can reduce the production of cholesterol 7α-hydroxylase, thereby reducing the production of bile acids, causing cholesterol to accumulate in cells. At the same time, up-regulating the expression of the vascular cellular adhesion molecule-1 (VCAM-1) can promote monocyte adhesion, activate protein kinase C (PKC) and p-NF-κB, and further lead to the formation of atherosclerotic plaque (Ma et al., 2017), thus increasing the risk of cerebrovascular events. Furthermore, a prospective cohort study also confirmed that an elevation in inflammation-associated monocytes caused by elevated TMAO levels can raise the risk of stroke and compromise the severity of stroke (Zhu et al., 2018, 2021). At the same time, TMAO can also reflect the human gut microbiota, which suggests that we can reduce the risk of cerebrovascular diseases and the prognosis of adverse cerebrovascular disorders by regulating the gut microbiota. For instance, we can prevent and treat cerebrovascular diseases by regulating TMAO levels by controlling the composition of TMAO-related bacteria in the gut microbiota. Probiotic preparations specifically tailored for this purpose are expected to form the foundation of treatment strategies for cerebrovascular diseases.

Furthermore, the crucial involvement of SCFA in stroke has also gained a high profile recently. It was observed that hemorrhagic transformation (HT), a life-threatening stroke complication in MCAO rats, correlated with inflammatory response and serum levels of SCFA (Huang Q. et al., 2022). They found that the total SCFA, specifically butyrate and valeric acid, was significantly lower in HT rats than in non-HT rats. At the same time, SCFA has also been linked to stroke treatment. Studies have shown that SCFA levels in ischemic stroke rats are reduced, and it has been shown that ischemic stroke can be effectively treated by transplanting SCFA-rich feces and supplementing it with butyric acid (Chen et al., 2019). Interestingly, Lee et al. (2020) also found that transplanting feces containing higher SCFA levels or related bacteria could effectively alleviate nerve defects and inflammation after stroke in elderly male mice, and promote post-stroke recovery in elderly mice. At the same time, studies have shown that SCFA can promote post-stroke recovery by altering the recruitment of brain-resident immune cells in the brain (Sadler et al., 2020). By increasing the level of systemic SCFA, it is expected to be applied to clinical diagnosis and treatment to improve the poor prognosis of stroke patients.

Some studies have shown the relationship between TBA levels and the severity and prognosis of acute ischemic stroke (AIS). Huang L. et al. (2022) found that TBA levels in patients admitted to the hospital with AIS were inversely associated with mortality within three months. Moreover, another research showed that higher TBA was associated with smaller hematoma volume and lower clinical severity (Wang K. et al., 2018). Therefore, serum TBA levels are likely to play a protective role in the severity and poor prognosis of ischemic stroke. Lowering serum TBA levels through diet and medications may predict lower mortality and fewer stroke sequelae in stroke patients. In addition, some current studies have also confirmed that conjugated and unconjugated bile acids are related to the occurrence and development of stroke. In conjugated bile acids, a clinical trial has shown that higher concentrations of deoxycholic acid (DCA), lithocholic acid (LCA), and cholic acid (CA) in feces in stroke patients are associated with higher survival after stroke. They also found that decreased bile acid excretion (BAE) may be an independent risk factor for stroke (Charach et al., 2020). For stroke severity or morbidity, Bian et al. (2019) found that DCA could improve acute cerebral infarction (ACI) induced nerve damage by inverting the Nrf2 signaling pathway. In unconjugated bile acids, a study of metabolite analysis in young stroke patients found that Glycochenodeoxycholic acid (GCDCA) concentrations were significantly higher in the stroke group than healthy controls (Liu et al., 2021). Besides, Wu et al. (2020) have demonstrated that TUDCA could attenuate neuronal apoptosis and improve neurological functions through TGR5/ SIRT3 signaling pathway after spontaneous subarachnoid hemorrhage (SAH). Interestingly, another research also has shown that TUDCA enhanced cerebral blood flow, reduced BBB permeability, inhibited the ER stress through the PERK/eIF2α/ATF4/CHOP signaling pathway, blocked the Caspase-12-dependent ER-stress mediated apoptosis, resulting in significantly improved neurological function of mice subjected to SAH (Chen X. et al., 2020). Accordingly, TUDCA is expected to be the first-line anti-apoptosis drug for SAH patients and reduce the related neurological sequelae. These results all suggest that BA is likely to have the potential to predict stroke outcomes in stroke patients.

Intestinal dysbacteriosis is also closely related to stroke treatment and prognosis. A recent study has identified a new way of regulating the GBA. Benakis et al. (2016) have shown that intestinal dysbacteriosis affects the outcome of ischemic stroke by altering dendritic cell activity and immune homeostasis in the small intestine, leading to an increase in regulatory T cells and a decrease in IL-17γδ T cells. Their findings shed new light on the immune mechanisms of stroke. Studies by Ling et al. (2020) suggest that Enterobacteriaceae, in particular, may be able to predict post-stroke cognitive impairment (PSCI), a common neuropsychiatric complication of stroke, while being used as a clinical biomarker for PSCI. For the treatment of stroke, several studies have pointed out that increasing the intake of SCFA can play a therapeutic role in stroke mice (Chen et al., 2019; Lee et al., 2020). In addition, Benakis et al. (2020b) have shown that mice treated with antibiotic cocktails significantly reduce infarct volume in the acute phase of stroke after changing the gut microbiota while improving neuromotor function in mice. Consequently, this evidence demonstrated the importance of the gut microbiota in the short-time and long-term outcomes of ischemic stroke. At the same time, microbiome-targeted therapies related to specific microbial enzymatic pathways may provide a better prognosis for patients at high risk of stroke. It has also been proposed to regulate the composition of the gut microbiota by oral administration of specific probiotics or by fecal microbiota transplantation (FMT) and to treat ischemic stroke by increasing beneficial metabolites such as SCFAs (Ling et al., 2020). Klimiec et al. (2016) observed that plasma endotoxin activity rises during ischemic stroke and is associated with worse short-term outcomes. Another research has also shown that metabolic endotoxemia can promote neuroinflammation after focal cerebral ischemia (Kurita et al., 2020). Therefore, the application of antibiotics against endotoxemia may be a new treatment strategy to improve the outcome of stroke. However, it should be noted that long-term use of antibiotics may lead to drug resistance. Studies by Tang et al. (2013) showed that plasma TMAO levels decreased significantly after taking broad-spectrum antibiotics to inhibit gut microbiota, and then increased again after stopping treatments. The advent of FMT has brought new hope (Wang et al., 2019; Zhang W. et al., 2020) for treating various diseases, which can effectively avoid intestinal dysbacteriosis caused by antibiotic treatment. In the future, whether antibiotics and FMT can be considered for treating stroke will be an exceedingly exciting research direction. At the same time, immunotherapy for the intestinal mucosal barrier also provides new ideas for treating stroke patients in the future.

### 4.2. Cerebrovascular malformation

Cavernous angiomas (CAs) are characterized by dysmorphic dilated vascular capillaries, or caverns, lined by endothelium (Yin et al., 2015; Zhu Y. et al., 2020). Cavernous hemangiomas (CCMs) are relatively common cerebrovascular malformations and a common clinical cause of hemorrhagic strokes and seizures (Spiegler et al., 2018). CCMs arise due to loss of function mutations in three genes, KRIT1 (aka CCM1), CCM2, and PDCD10 (aka CCM3), that encode components of a single, heterotrimeric, adaptor protein complex (Tang et al., 2019). The current standard treatments for CCMs are still symptomatic and surgically resected. Unfortunately, there is no specific drug for CCMs (Akers et al., 2017). Recent studies have shown that lipopolysaccharides (LPS) from Gram-negative bacteria (GNB) in the gut microbiome can drive the development of CCM disease by activating TLR4 and MEKK3 signaling in brain endothelial cells (Tang et al., 2017). Their study confirmed the central role of the gut microbiome and endothelial response to GNB in the pathogenesis of CCMs while demonstrating that the gut microbiome is the primary source of TLR4 ligands needed to stimulate CCMs formation in mice.

The minor differences in gut microbiota may significantly impact the progression of CCMs disease in this animal model. Previous studies have hypothesized the existence of the CCMs’ gut-brain axis (Tang et al., 2017). Interestingly, the study by Tang et al. (2019) further demonstrated the presence of the CCMs gut-brain axis while identifying a central molecular component of the gut-brain axis in CCMs disease: the colonic mucus barrier. They concluded that the down-regulation of PDCD10 signaling in the brain endothelium and intestinal epithelium led to CCMs in mouse models. Surprisingly, their study also found that dexamethasone effectively inhibited the formation of CCMs in mice due to the combined action of brain endothelial cells and intestinal epithelial cells. Therefore, the activity of dexamethasone is probably based on its multiple critical molecular and cellular mechanisms in targeting CCMs’ gut-brain axis. The recent research based on 16S rRNA gene sequencing technology, confirmed that CCMs patients have a unique gut microbiome, and LPS synthesis-related genes are more abundant in CA patients, consistent with intestinal LPS in driving CCMs disease (Polster et al., 2020). The study further demonstrated that CCMs patients with different disease characteristics have different gut microbiota, and the combination of plasma biomarkers and gut microbiome validated this idea. Future research can target gut microbiota and CCMs brain-gut axis-related targets to provide new strategies for treating CCMs. At the same time, combining the microbiome and circulating factors may also serve as biomarkers of potential disease severity and prognosis, providing new ideas for diagnosing CCMs. However, it should be noted that drugs targeting CCMs must fully consider the potential impact on the intestinal mucosal barrier function. Future research on targeted drugs should take more into account the existence of the intestinal mucosal barrier to effectively reduce the toxic side effects of targeted drugs.

### 4.3. Intracranial aneurysm

Intracranial aneurysm (IA) refers to the limitation and pathological expansion of the intracranial artery wall, which has emerged as the leading cause of SAH due to the risk of rupture (Macdonald and Schweizer, 2017). SAH caused by intracranial aneurysm rupture has the characteristics of a large number of occurrences, a wide range, and poor prognostic outcome. It has become a cerebrovascular disease that seriously endangers human health (Connolly et al., 2012). Despite extensive research in recent years, the exact mechanisms that lead to the pathogenesis of IAs are poorly understood. Therefore, there is an urgent need to find ways to diagnose and treat intracranial aneurysmal SAH to improve its poor prognosis.

Currently, the pathogenesis of IA is not completely clear, but the current evidence has confirmed that inflammation plays a significant role in it (Berge et al., 2016; Fennell et al., 2016). Recent research suggests that IA is partly caused by hemodynamically triggered endothelial cell dysfunction. This is followed by an inflammatory response of the vessels, accompanied by an increase in the activity of the inflammatory transcription factor NF-κB (Wei et al., 2011). The inflammatory response stimulates the phenotypic modulation of vascular smooth muscle cells (VSMCs) from a contractile to a pro-inflammatory/pro-matrix remodeling phenotype, followed by their degeneration, which may be crucial to IA formation and progression (Owens, 2007). At the same time, the gut microbiota also plays a crucial role in the development of many diseases through inflammation (Zhao et al., 2021; Cai et al., 2022). Therefore, the gut microbiota is also closely related to the occurrence and development of IAs. The findings of Shikata et al. (2019) are the first direct confirmation that the gut microbiota can influence the pathophysiology of IAs by modulating local inflammation. They found that antibiotics can reduce the effect of inflammation of cerebral arteries during IA formation and thus effectively reduce the formation of IAs. Metagenome-wide association studies (MWAS) performed serum metabolomics analysis of patients with IAs for the first time to identify microbial species associated with the unruptured intracranial artery (UIA), and further explored their effects on host amino acid and fatty acid metabolism (Shikata et al., 2019; Li et al., 2020). They reconfirmed the possible causal relationship between changes in the gut microbiota of UIA patients and more vital systemic inflammation. They also found that taurine can protect mice from the formation and rupture of IAs, while taurine supplementation can also reverse the progression of IAs. Not only does this study provide a new idea for the diagnosis and treatment of intracranial aneurysms, but it also shows that gut microbial metabolites may also impact the rupture of aneurysms. Another study provided new perspectives on intracranial ruptured aneurysm (Kawabata et al., 2022). Using 16S rRNA sequencing technology, they conducted a multicenter, prospective case–control study. For the first time, the relationship between gut microbiota dysregulation and intracranial rupture aneurysm has been elucidated: the gut microbiota characteristics of patients with the UIA and ruptured intracranial artery (RA) are significantly different. In addition, Campylobacter and Corynebacterium may be associated with intracranial brain aneurysm rupture. Surprisingly, they also elucidated for the first time the mechanism by which Campylobacter infection leads to the rupture of intracranial aneurysms, demonstrating its close association with inflammation and the MMP family. Finally, they concluded that Campylobacter could promote vascular remodeling and cell death of the cerebral artery wall by increasing inflammation-related cytokines, neutrophil-derived proteolysis, and oxidative stress. At the same time, it can finally lead to the rupture of IAs through the effects of hemodynamics and genetics. However, the current research on IAs and gut microbiota is still highly challenging. The diversity of gut microbiota is closely related to the environment, and the composition of gut microbiota in different regions is diverse. For example, one study reported that the gut microbiota of the Japanese population is exceedingly different from other populations (Park et al., 2021). Therefore, the current research may have specific limitations. In the future, we need to expand the scope of study further to understand other gut microbiota and the occurrence and development of IAs (Table 1).

**Table 1:** Gut microbiome metabolites in cerebrovascular diseases.

Metabolite		Associated bacteria	Research progress in cerebrovascular diseases
Short-chain fatty acids	Acetic acid	Anaerobic	Butyrate suppresses the production of pro-inflammatory cytokines, such as TNF-α, IL-12, and IF-γ, and upregulates the production of anti-inflammatory IL-10 by monocytes *in vitro* (Säemann et al., 2000).
	Propionic acid	Bacillus	SCFA can prevent vascular inflammation by activating GPR41/43 and inhibiting HDAC (Verhaar et al., 2020).
	Isobutyric acid	Bifidobacterium	Total SCFAs, especially butyrate and valeric acid, were significantly lower in the cecal contents of HT rats than in those of non-HT rats (Huang Q. et al., 2022).
	Butyric acid	Eubacteria	SCFA content in the blood of ischemic stroke rats decreased, and ischemic stroke can be effectively treated by transplanting feces rich in SCFA and supplementing butyric acid (Chen et al., 2019).
	Isovaleric acid	Streptococcus	Transplanting feces containing higher SCFA level or related bacteria can effectively alleviate the nerve defects and inflammation of old male mice after stroke, and promote the recovery of old mice after stroke (Lee et al., 2020).
	Valeric acid	lactobacillus	SCFAs modulate poststroke recovery *via* effects on systemic and brain resident immune cells (Sadler et al., 2020).
Choline metabolites	Methylamine	*F. prausnitzii*	TMAO induces vascular inflammation by activating the NLRP3 inflammasome through the SIRT3-SOD2-mtROS signaling pathway (Chen et al., 2017).
	Dimethylamine	Bifidobacterium	TMAO can up-regulate leukocyte recruitment and the expression of pro-inflammatory cytokines IL-1β, IL-18 and TNF-α, and reduce the expression of anti-inflammatory cytokine IL-10 (Chen et al., 2017).
	Trimethylamine		TMAO promotes early pathological process of atherosclerosis by accelerating endothelial dysfunction, including decreasing endothelial self-repair and increasing monocyte adhesion (Ma et al., 2017).
	Betaine		Elevated circulating TMAO during the aging process may deteriorate EC senescence and vascular aging, which is probably associated with repression of SIRT1 expression and increased oxidative stress (Ke et al., 2018).
	Trimethylamine N-Oxide		High levels of TMAO in the blood of stroke patients affect stroke severity (109).
			The increase of monocytes related to inflammation caused by the increase of TMAO level will lead to the increase of the risk of stroke (Zhang X. et al., 2020).
			TMAO has been confirmed to directly cause platelet hyperreactivity and enhance thrombosis, thereby increasing the risk of cardiovascular accidents (Zhu et al., 2016).
			Higher TMAO level is associated with increased risk of first stroke. Patients with low folate and high TMAO had the highest rate of stroke (Nie et al., 2018).
Bile acids	Conjugated bile acids:	Lactobacillus	Deoxycholic acid, cholic acid and lithocholic acid was higher in stroke-free patients compared to those who developed stroke (Quigley, 2017).
	CA, CDCA	Bifidobacterium	Decreased bile acid excretion is an independent risk factor for stroke (Charach et al., 2020).
	DCA, LCA	Enterobacter	Compared with the healthy control group, GCDCA concentration in stroke group was significantly higher (Liu et al., 2021).
	Conjugated bile acids:	Bacteroides	DCA can improve the nerve injury induced by acute cerebral infarction by reversely regulating Nrf2 signal pathway (Bian et al., 2019).
	GCA, TCA	Clostridia	The TBA level of AIS patients was negatively correlated with the mortality within 3 months (Huang L. et al., 2022).
	GCDCA, TCDCA		TUDCA could attenuated neuronal apoptosis and improve neurological functions through TGR5/SIRT3 signaling pathway after SAH (Wu et al., 2020).
			TUDCA improved cerebral blood flow, reduced BBB permeability, inhibited the ER stress through the PERK/eIF2α/ATF4/CHOP signaling pathway, blocked the Caspase-12-dependent ER-stress mediated apoptosis, resulting in significantly improved neurological function of mice subjected to SAH (Chen X. et al., 2020).
Lipopolysaccharide		Bifidobacterium	The lipid A component of LPS is the main PAMP, which can interact with TLR4 (Ciesielska et al., 2021).
		Klebsiella	LPS forms a complex with LBP binding protein, and bind to CD14 on monocytes. This may lead to the production of pro-inflammatory cytokines, such as TNF-α, IL-1 and IL-6 (Sun et al., 2016).
		Enterobacter	An increased plasma LPS level constitutes a substantial risk factor for incident carotid atherosclerosis (Macrez et al., 2011).
		Citrobacter	Plasma endotoxin activity rises during ischemic stroke and is associated with worse short-term outcome (Klimiec et al., 2016).
		Clostridium	Metabolic endotoxemia can promote neuroinflammation after focal cerebral ischemia (Kurita et al., 2020).
			Ex secreted from the LPS-stimulated macrophage RAW264.7 cell line (LPS-Ex) is effective in generating anti-inflammatory and neuroprotective effects by enhancing the microglial M2 polarization (Zheng et al., 2019).

### 4.4. Gut microbiota and metabolites in other vascular diseases

#### 4.4.1. Pulmonary hypertension

Pulmonary Hypertension (PH) is a progressive and devastating disease characterized by pulmonary artery pressure greater than 25 mmHg. The leading cause of death was a right ventricular failure (Oliveira et al., 2020). Several studies have shown a close connection between PH and gut microbiota. The concept of the lung-gut axis (Wypych et al., 2019) has also promoted the research progress of PH. Hong et al. (2021) used a multinomial approach to study the correlation between the gut microbiota and host metabolome in PH and NPS 2143-treated rats, confirming changes in the gut microbiome in rats with PH. At the same time, there are differences between gut microbial metabolites in PH patients and ordinary people.

Interestingly, a recent study has discovered the association between TMAO and PH, finding that circulating TMAO was elevated in high-risk PH patients compared with healthy controls or low-risk PH patients. The use of 3,3-Dimethyl-1-butanol (DMB) significantly reduced right ventricular systolic blood pressure and the degree of pulmonary arterial muscularization in PH rats by reducing the content of TMAO (Huang Y. et al., 2022). At the same time, it was clarified that the reduction of TMAO can decrease the formation of pulmonary arterial muscularization by inhibiting the production of chemokines and cytokines and ultimately delaying the occurrence of PH. These findings deepen our understanding of the gut microbiota and PH, as well as confirm the existence of the gut-lung axis.

#### 4.4.2. Portal hypertension

Portal hypertension is a pathological condition associated with liver injury, most commonly precipitated by cirrhosis. As the pressure in the portal vein rises, many fatal complications occur. Typically, the gut microbiome coordinates with the liver to maintain homeostasis in the body, and the concept of the gut–liver axis was born (Huang et al., 2021). Current research has confirmed that changes in the gut microbiota, as well as the intestinal mucosal barrier, may influence the degree of hepatic steatosis, inflammation, and fibrosis through multiple interactions with the host immune system and other cell types, leading to changes in portal venous pressure and ultimately influencing the progression of cirrhosis (Henao-Mejia et al., 2012). PAMP is the bacterial endotoxin known as lipopolysaccharide (LPS) in the outer membrane of gram-negative bacteria. The current study found that intraperitoneal injection of LPS has increased portal venous pressure (Steib et al., 2010), while increasing intestinal permeability. In addition, bacterial translocation, endotoxemia, and pro-inflammatory cytokines have been found to impair the contractility of mesenteric vessels in patients with cirrhosis and thereby increase portal venous pressure (Arab et al., 2018). For the treatment of portal hypertension, there are also some surprising results for the gut microbial metabolites BA. Regulation of BA nuclear receptors with the potent, selective FXR agonist Ocaliva (OCA) has improved portal hypertension through two different pathways. In both models, OCA therapy has been shown to reactivate signaling pathways downstream of FXR and reduce portal pressure by reducing intrahepatic total vascular resistance without developing systemic hypotension (Verbeke et al., 2014). Additionally, OCA has been shown to reduce bacterial translocation and reduce intestinal inflammation in rats with ascites cirrhosis (Úbeda et al., 2016). Therefore, the regulation of BAs signaling may be a new target for portal hypertension regulation in the future, closely related to the gut microbiota.

#### 4.4.3. Vasculitis

Vasculitis refers to the infiltration of inflammatory cells in and around the blood vessel wall, accompanied by vascular endothelial cell injury, including cellulose deposition, collagen fiber degeneration, and endothelial cell and muscle cell necrosis. Many studies have shown the relationship between gut microbiota and vasculitis. Wang X. et al. (2018) first found that gut microbiota dysbiosis is associated with Henoch-Schönlein purpura (HSP) in children. The populations of Parabacteroidota and Enterococcus increased significantly in the gut microbiota of HSP patients, emphasizing the significance of gut microbiota dysbiosis in HSP. At the same time, Li et al. (2021) also found that the abundance of gut microbiota in children with IgA vasculitis was lower than that of normal children. Metabolomics has found that Bacteroidota, Bacillota, Proteus, and Actinomycetes are the four most abundant bacteria in children’s gut microbiota. Pseudomonadota and actinomycetes have also been shown to be associated with organ involvement in IgA vasculitis. Similarly, with the deepening of research, other vasculitides, such as Kawasaki disease and Behcet’s disease, have been confirmed to be related to the gut microbiota (Chen J. et al., 2020; Ma et al., 2021). Shortly, it is hoped that the field of the gut microbiome can be applied to the treatment of vasculitis, and the gut microbiota can be used as a biomarker to facilitate the early diagnosis and prognosis assessment of vasculitis diseases.

#### 4.4.4. Summary

**Figure 2:**
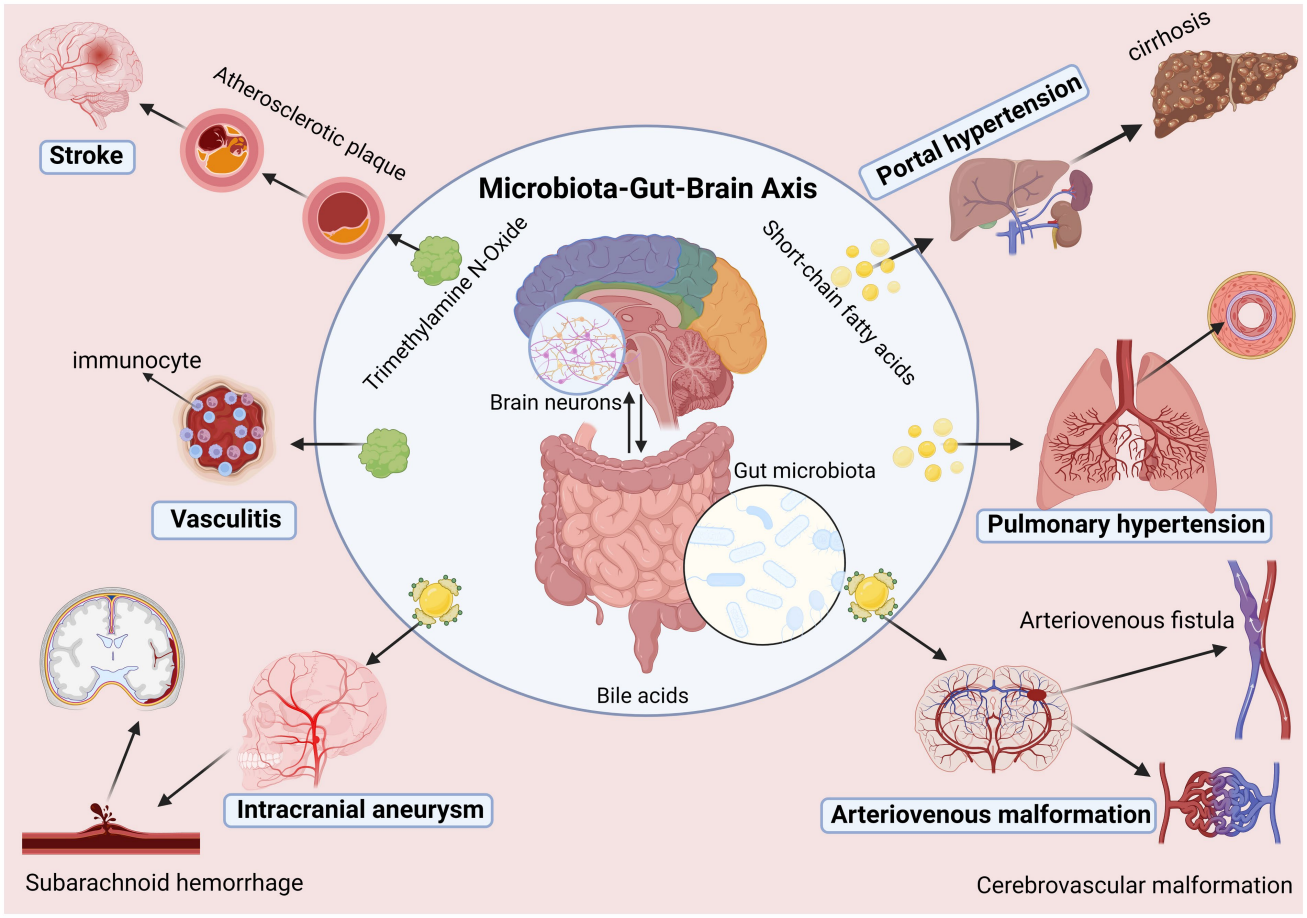
Microbiota-gut-brain axis is involved in the occurrence and development of cerebrovascular diseases.

In addition, some recent studies have also found specific correlations between some other vascular diseases and gut microbiota. For example, it has been found that the composition of the gut microbiota in patients with diabetic angiopathy is significantly different from that of ordinary people (Iatcu et al., 2021). Disappointingly, although our understanding of the previous interaction between the gut microbiota and the host has deepened in recent years, we still need a comprehensive understanding of the molecular mechanism of the GBA. At the same time, there are significant individual differences in gut microbiota itself, and there are differences in age, race, and sex that also limit the progress of related research (Bibbò et al., 2016; Takagi et al., 2019). In addition, current research is still blank for some vascular diseases such as moyamoya disease, arteriovenous fistula, and functional vascular diseases. However, it is undeniable that the recent research on gut microbiota and vascular diseases shows that a deeper understanding of gut microbiota can help us understand cerebrovascular diseases and also help us diagnose and treat cerebrovascular diseases, an exceedingly gratifying discovery (Figure 2).

## 5. Discussion

Cerebrovascular disease has high morbidity, disability rate, and mortality. Therefore, heart disease and malignant tumors constitute the three major causes of human death (Caprio and Sorond, 2019). Research on the gut microbiota and cerebrovascular disease has provided new insights into the effective prevention and treatment of cerebrovascular disease, thus reversing the traditional recognition of cerebrovascular disease and neuroinflammation. Although our research on the interaction between the gut microbiome and cerebrovascular diseases is still in its infancy, the results of various research results that continue to emerge are still surprising, especially the role of specific intestinal flora and its metabolites can delay the occurrence and progression of cerebrovascular diseases.

Currently, some new treatment strategies, such as FMT and phage therapy (Wang et al., 2019; Federici et al., 2022), can improve intestinal dysbacteriosis through probiotics, dietary intervention and other ways to treat cerebrovascular diseases, which have potential research value. Several studies using CeVD animal models have confirmed the role of FMT in the occurrence and treatment of cerebrovascular diseases. Intestinal T cells develop protective activity following transplantation of feces from a young population into mice with IS. Treg cells and IL-17 T cells contribute to decreased inflammation, neurological deficits, and impairment of intestinal barrier function following stroke (Lee et al., 2020; Haak et al., 2021; Zou et al., 2022). In mice with ICH, transplantation of bacterial flora can affect T cells in the brain, reduce neuroinflammation following bleeding, and restore the average fluorescence intensity of the tight junction proteins occludin and claudin-1, thereby restoring intestinal barrier function (Wang et al., 2021). At the same time, as FMT increases the possibility of antibiotic-resistant bacterial infections, the advent of phage therapy could better address antibiotic resistance. The combination of FMT and Phage therapy in patients with cerebrovascular disease complicated with multiple drug-resistant infections caused by prolonged bed rest may better improve their poor prognosis and reduce the incidence of complications. In the future, targeted agents against the gut microbiota can be applied in a simple and non-invasive manner to the clinical diagnosis and treatment of cerebrovascular diseases. However, the current research on gut microbiota also has some limitations. The pathological state of stroke will inhibit the body’s immune ability, thereby enhancing intestinal permeability and promoting microbiota translocation. The potential for transmission of antibiotic-resistant pathogens *via* FMT is dramatically increased, ultimately leading to fatal sepsis. A recent review of FMT safety found that serious adverse events occur in 2–6% of patients, depending on the route of administration (Wang et al., 2016). A uniform standard for screening and selection of FMT-related donors, fluid preparation and transplantation procedures are needed to effectively reduce the risk of potential infection, which is especially relevant for immunodeficient patients. In addition, most studies have been conducted on rodents and lack sufficient evidence of efficacy and long-term safety and evidence-based medicine (Walter et al., 2020; Gheorghe et al., 2021). FMT has inconsistent treatment outcomes due to differences in the route of administration (Ng et al., 2020). Therefore, the selection of the proper and efficacious method of administration is also a problem currently encountered. Consequently, we must be very cautious in analyzing and studying the impacts of gut microbiota on human beings. How to make a specific gut microbiota successfully target and colonize the human intestine will also become a problem that needs to be solved in the future.

This review summarizes the relevant research on the gut microbiome and cerebrovascular diseases in recent years, showing the close relationship between gut microbiota and cerebrovascular diseases. At the same time, we also elaborated on the relevant molecular mechanisms of the existing gut microbiota and its metabolites causing the occurrence and development of cerebrovascular diseases. However, the specific molecules, locations, and mechanisms acting on cerebrovascular diseases after intestinal dysbacteriosis still need to be further explored, which will become a research hotspot in the future. Due to some of the above limitations, the biomarkers of gut microbiota and its metabolites for early diagnosis, prognosis, and therapeutic targets of cerebrovascular diseases still need further more accurate and comprehensive research.

## Author contributions

XL, WW, and XW proposed the ideas and drafted the outlines. HX, ZX, and SL performed the literature search and completed the manuscript. ZL, XH, JJ, and QZ helped revise the manuscript and provided support in need. All authors contributed to the design and writing of the manuscript.

## Funding

This work was supported by the Climbing Project for Medical Talent of Zhongnan Hospital, Wuhan University.

## Conflict of interest

The authors declare that the research was conducted in the absence of any commercial or financial relationships that could be construed as a potential conflict of interest.

## Publisher’s note

All claims expressed in this article are solely those of the authors and do not necessarily represent those of their affiliated organizations, or those of the publisher, the editors and the reviewers. Any product that may be evaluated in this article, or claim that may be made by its manufacturer, is not guaranteed or endorsed by the publisher.
